# Phase Transitions Drive the Formation of Vesicular Stomatitis Virus Replication Compartments

**DOI:** 10.1128/mBio.02290-17

**Published:** 2018-09-04

**Authors:** Bianca S. Heinrich, Zoltan Maliga, David A. Stein, Anthony A. Hyman, Sean P. J. Whelan

**Affiliations:** a Department of Microbiology and Immunobiology, Harvard Medical School, Boston, Massachusetts, USA; b Max Planck Institute for Cell Biology and Genetics, Dresden, Germany; c Laboratory of Systems Pharmacology, Harvard Medical School, Boston, Massachusetts, USA; d Department of Biomedical Sciences, Oregon State University, Corvallis, Oregon, USA; Icahn School of Medicine at Mount Sinai

**Keywords:** negative-strand RNA virus, phase separation, rhabdovirus, viral replication, viroplasm, virus-host interactions

## Abstract

RNA viruses that replicate in the cell cytoplasm typically concentrate their replication machinery within specialized compartments. This concentration favors enzymatic reactions and shields viral RNA from detection by cytosolic pattern recognition receptors. Nonsegmented negative-strand (NNS) RNA viruses, which include some of the most significant human, animal, and plant pathogens extant, form inclusions that are sites of RNA synthesis and are not circumscribed by a membrane. These inclusions share similarities with cellular protein/RNA structures such as P granules and nucleoli, which are phase-separated liquid compartments. Here we show that replication compartments of vesicular stomatitis virus (VSV) have the properties of liquid-like compartments that form by phase separation. Expression of the individual viral components of the replication machinery in cells demonstrates that the 3 viral proteins required for replication are sufficient to drive cytoplasmic phase separation. Therefore, liquid-liquid phase separation, previously linked to organization of P granules, nucleolus homeostasis, and cell signaling, plays a key role in host-pathogen interactions. This work suggests novel therapeutic approaches to the problem of combating NNS RNA viral infections.

## Abstract (precis)

RNA viruses compartmentalize their replication machinery to evade detection by host pattern recognition receptors and concentrate the machinery of RNA synthesis. For positive-strand RNA viruses, RNA replication occurs in a virus-induced membrane-associated replication organelle. For NNS RNA viruses, the replication compartment is a cytoplasmic inclusion that is not circumscribed by a cellular membrane. Such structures were first observed in the cell bodies of neurons from humans infected with rabies virus and were termed Negri bodies. How the replication machinery that forms this inclusion remains associated in the absence of a membrane has been an enduring mystery. In this article, we present evidence that the VSV replication compartments form through phase separation. Phase separation is increasingly recognized as responsible for cellular structures as diverse as processing bodies (P-bodies) and nucleoli and was recently demonstrated for rabies virus. This article further links the fields of host-pathogen interaction with that of phase separation.

## INTRODUCTION

For viruses with RNA genomes, those that carry out RNA synthesis in the cytoplasm outnumber those that do so in the nucleus by about 10:1. RNA viruses typically establish specialized organelles in the cytoplasm in which RNA synthesis occurs (1, 2). The nonsegmented negative-strand (NNS) RNA virus vesicular stomatitis virus (VSV) is capable of transcribing its genomic RNA initially in the absence of any apparent specialized replication compartment (3). By 4 h postinfection (hpi), however, electron-dense structures appear in the cytoplasm, which are enriched in proteins required for RNA synthesis, namely, the nucleocapsid protein (N), the phosphoprotein (P), and the multifunctional large protein (L). Once formed, these compartments become the major sites of RNA synthesis (3–5). Accordingly, the viral components of the replication machinery, namely, N, P, and L, are targeted to inclusions within infected cells. Similar structures have been observed in cells infected with related viruses, including rabies virus (RABV [4, 5]), Ebola virus (6), and measles virus (7). Ultrastructural analyses of infected cells reveal that no membrane boundary separates the inclusions from the surrounding cytoplasm (3–5, 8). This raises the question of how the machinery necessary for RNA synthesis is contained within this viroplasm.

The 11,161-nucleotide genomic RNA of VSV is found completely encased within a sheath of 1,240 N protein molecules (9). This protein-RNA complex (N-RNA) is used as the template by L for transcription of the viral mRNAs and for RNA replication. The L protein, however, is unable to efficiently engage the N-RNA alone. Instead it depends upon an accessory cofactor, the P protein, which binds L through its N terminus and the template-associated N via its C terminus (10–12). This N-RNA:P-L complex is sufficient for transcription of viral mRNA *in vitro* (13, 14). Replication of the genomic RNA depends also on recognition of the N-RNA by an L-P complex, but additionally requires the recruitment of newly synthesized soluble N protein to bind the nascent replication product. The P protein is also required to keep N soluble when not bound to the viral genomic RNA, and this is accomplished by a region at the N terminus of P (15). An oligomerization domain located within the center of the P protein is thought to facilitate the simultaneous binding of the polymerase and the N-RNA (9, 10, 16–18). The remainder of P is intrinsically disordered (19). Although the interactions among the various components of the RNP have been mapped, knowledge of how these individual components contribute to the formation of the viroplasm is lacking.

Liquid phase separation is a mechanism for generating cellular compartments without the need for a membrane (20–23). During this process, cytoplasmic or nuclear components condense into a distinct phase that exhibits classic fluid properties. Transient, multivalent macromolecular interactions drive phase separation *in vitro* (8), and physiological inputs are coupled to condensation of complex mixtures during cell stress (23) or to control the formation of RNA-protein complexes in response to developmental cues (8, 21). The three key properties of a liquid phase that can be measured in cells are (i) fusion of two or more drops into a single drop (22), (ii) a round shape induced by surface tension, and (iii) rapid internal diffusion. In the present study, we therefore sought to test whether these properties are shared by VSV viroplasm. We provide evidence in support of all 3 properties for the VSV viroplasm and demonstrate that the viral N, P, and L proteins are necessary for their formation. We also show that the viral genomic RNA, or the catalytic activity of the L-encoded RNA-dependent RNA polymerase (RdRp), is not required for formation of this phase-separated structure. These data demonstrate that formation of the VSV viroplasm is an intrinsic property of the viral N, P, and L proteins.

## RESULTS

### Replication compartments are highly dynamic undergoing fission and fusion.

**FIG 1:**
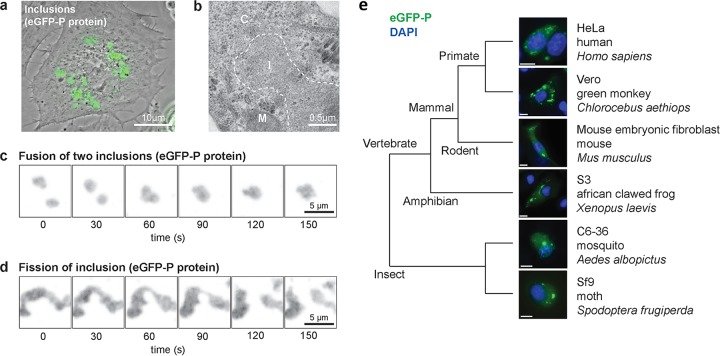
Fluid-like properties of the non-membrane-associated VSV replication compartment. (a) Composite phase-contrast and laser micrograph of Vero cells infected with rVSV-eGFP-P 6 h postinfection (6 hpi). Scale bar, 10 µm. (b) Electron micrograph of rVSV-eGFP-P-infected Vero cells (6 hpi) showing electron-dense inclusion (I), mitochondrion (M), and cytoplasm (C). Scale bar, 0.5 µm. (c and d) Time-lapse GFP images (Movie 1) showing fusion (c) and fission (d) of viral inclusions in rVSV-eGFP-P-infected Vero cells (6 hpi). Arrow marks the clearly separated inclusion. Single-plane GFP fluorescence micrographs were captured every 30 s. Scale bars, 5 µm. (e) Phase-separated compartments form in cells from a diverse range of hosts. Shown are fluorescence micrographs of the following cell lines infected with rVSV-eGFP-P and counterstained for DNA: Homo sapiens HeLa S3 (6 hpi), Chlorocebus aethiops Vero (6 hpi), Mus musculus mouse embryonic fibroblast (8 hpi), Xenopus laevis S3 (8 hpi), Aedes albopictus C6-36 (48 hpi), and Spodoptera frugiperda Sf-9 cells (48 hpi). Scale bars, 10 µm.

Fusion of the amino terminus of the VSV phosphoprotein to enhanced green fluorescent protein (eGFP) serves to mark discrete cytoplasmic structures (Fig. 1a) previously defined as sites of viral RNA synthesis. Examination of infected cells by negative-stain electron microscopy confirms previous observations that no membrane boundary separates the inclusions from the surrounding cytoplasm (Fig. 1b). Those inclusions form in a broad range of cells, including those from insects, amphibians, and mammals (Fig. 1e). This result demonstrates that the information necessary for viroplasm formation and maintenance is virally encoded and that if host factors are required for their formation and maintenance, they are broadly conserved between different species (Fig. 1e).

One key property of a liquid phase that can be measured in cells is fusion of two or more drops into a single drop (22). To determine whether the VSV replication site inclusions exhibit such fluid-like properties, we infected Vero cells with VSV-eGFP-P and visualized the infected cells by live-cell fluorescence microscopy. Real-time imaging revealed the fluorescence signal is concentrated in dynamic, motile cytoplasmic inclusions (see Movie S1 in the supplemental material). Collision of two inclusions yields a fusion product within minutes (Fig. 1d; Movie S1). Elongation of inclusions by intracellular pulling forces often results in two fission products whose new ends collapse into a rounded surface (Fig. 1E; Movie S1). Those properties of the viral inclusions show that they are dynamic organelles subject to deformation, fission, and fusion on rapid timescales.

10.1128/mBio.02290-17.5 MOVIE S1  Dynamics of VSV inclusions. Shown is a 3D maximum-intensity projection of Vero cells 4 hpi with rVSV-eGFP-P and imaged for 30 min (2 frames per min). Inclusions exhibit vigorous movement throughout the cytoplasm and frequently undergo fusion and fission events. Download MOVIE S1, MOV file, 3.9 MB. Copyright © 2018 Heinrich et al. 2018 Heinrich et al. This content is distributed under the terms of the Creative Commons Attribution 4.0 International license.

### Intrinsic surface tension of replication compartments.

**FIG 2:**
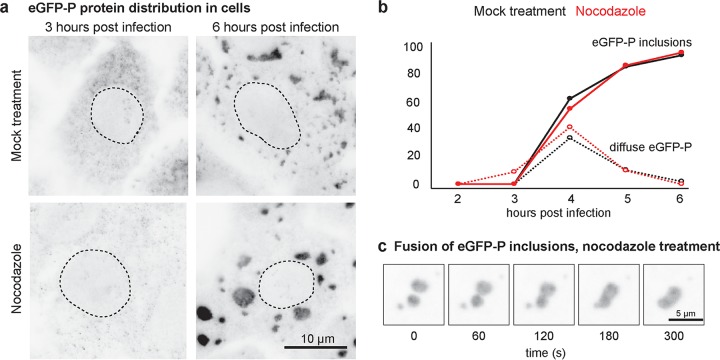
Replication compartment formation and maintenance are insensitive to microtubule depolymerization. (a) GFP fluorescence micrographs of Vero cells cultured in medium alone (mock treatment) or supplemented with nocodazole and fixed at 3 or 6 hpi with rVSV-eGFP-P. Cell nuclei are outlined for reference (dotted lines). Scale bar, 10 µm. (b) Time course for the appearance of eGFP in cytosol and viroplasm in Vero cells after infection with rVSV-eGFP-P and culture in medium alone (mock treatment) or supplemented with nocodazole. (c) Time-lapse GFP images (Movie S2) of fusion between viral inclusions in nocodazole-treated Vero cells infected with rVSV-eGFP-P and imaged every minute. Scale bar, 5 µm.

A second property exhibited by phase-separated structures is their ability to adopt a rounded shape induced by intrinsic surface tension. Evidence for intrinsic surface tension of the viroplasm is observed following the spontaneous separation of individual structures into two or more structures (Fig. 1e). To provide further support that the viroplasm adopts a round shape induced by intrinsic surface tension, we looked for ways to remove the intracellular forces that cause deformation and fission. We found that after disrupting the microtubule cytoskeleton with nocodazole, cells infected with VSV-eGFP-P display round inclusions that endure for several hours and are comparable to inclusions formed in mock-treated cells (Fig. 2a; see Movie S2 in the supplemental material). Furthermore, nocodazole treatment did not alter the kinetics of inclusion formation (Fig. 2b), and rounded inclusions fuse when brought together by random motion in cells (Fig. 2c; Movie S2). These observations support that rounding is an intrinsic property of the viroplasm and that microtubules are not required to establish or maintain such structures.

10.1128/mBio.02290-17.6 MOVIE S2  Dynamics of VSV inclusions in nocodazole-treated cells. Shown is a 3D maximum-intensity projection of Vero cells infected with rVSV-eGFP-P (4 hpi) and treated with nocodazole 1 h before imaging. Cells were imaged for 30 min (2 frames per min). Inclusions appear as rounded droplets that are generally still but retain the ability to undergo fusion. Download MOVIE S2, MOV file, 3.1 MB. Copyright © 2018 Heinrich et al. 2018 Heinrich et al. This content is distributed under the terms of the Creative Commons Attribution 4.0 International license.

### Viroplasm constituents exhibit rapid internal diffusion.

**FIG 3:**
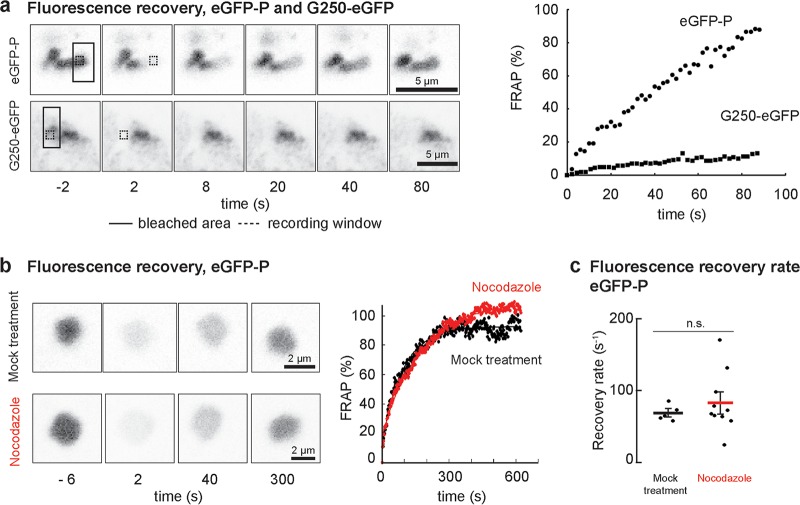
Rapid exchange of the components of the replication compartments. (a) Time-lapse images and quantification of FRAP experiments in Vero cells infected with rVSV-eGFP-P (filled circles) or transfected with a cDNA encoding G250-GFP (filled squares). The photobleached region (solid line box) and regions used for quantification (dotted line boxes) are indicated. Scale bars, 5 µm. (b) Time-lapse images and quantification of whole-inclusion FRAP in Vero cells infected with rVSV-eGFP-P. Integrated fluorescence intensity over time was fit to a single-exponential recovery curve for representative inclusions from mock-treated (black; *t* = 0.010 s^−1^; *R*^2^ = 0.979) or nocodazole-treated (red; *t* = 006 s^−1^; *R*^2^ = 0.993) cells. Scale bars, 2 µm. (c) FRAP recovery half-lives for inclusions in mock-treated (5 cells; mean, 68 s; standard deviation [SD], 11 s) and nocodazole-treated (10 cells; mean, 83 s; SD, 41 s) had no significant difference (unpaired *t* test, *P* = 0.3).

We next tested whether viral proteins present within the viroplasm undergo rapid internal diffusion. To do this, we took advantage of VSV-eGFP-P and a variant in which the L protein was engineered to contain eGFP inserted between the connector domain and methyltransferase domain. We compared fluorescence recovery after photobleaching (FRAP) of eGFP-P (see Movie S3 in the supplemental material) and L-eGFP in viral inclusions to that of aggresomes formed by G250, a GFP-tagged aggregation domain (see Movie S4 in the supplemental material). As expected, the signal in the G250-GFP aggresomes did not recover after photobleaching, consistent with their static aggregated nature. In contrast, we observed full and rapid recovery of eGFP fluorescence in the viroplasm of VSV-eGFP-P-infected cells (Fig. 3a). Rapid FRAP of L and P proteins was confirmed by measurements in cells infected with a dual red fluorescent protein (RFP)-P- and eGFP-L-labeled virus (see Fig. S1 in the supplemental material). Consistent with the larger size and lower abundance of L, its exchange was slightly slower than that of P.

10.1128/mBio.02290-17.1 FIG S1  L and P display rapid but distinct FRAP kinetics in viroplasm. (a) Visualization of RFP-P and eGFP-L images of an inclusion in Vero cells infected with rVSV (RFP-P and L-eGFP) before and after photobleaching (box). Scale bar, 5 µm. FRAP for RFP-P (filled circles) and eGFP-L (open circles) were measured as a pixel average (between short lines 0.5 µm from the bleach front) and fit to a recovery curve with single-exponential (RFP, 62%, *t* = 0.83 s^−1^; eGFP, 15%, *t* = 0.45 s^−1^) and linear (RFP, 38%, mean, 0.04 s^−1^; eGFP, 85%, mean, 0.04 s^−1^) components. *R* = 0.986 and 0.995 for eGFP and RFP, respectively. (b) FRAP quantification of L-eGFP and RFP-P in viroplasm. Download FIG S1, TIF file, 0.8 MB. Copyright © 2018 Heinrich et al. 2018 Heinrich et al. This content is distributed under the terms of the Creative Commons Attribution 4.0 International license.

10.1128/mBio.02290-17.7 MOVIE S3  Rapid exchange of eGFP-P in viral inclusions. Shown is partial photobleaching of a viral inclusion in Vero cells infected with rVSV-eGFP-P (4 hpi). Cells were imaged 3 frames before and 45 frames after photobleaching (28 frames per s). Rapid FRAP was observed on the right half of the inclusion. Download MOVIE S3, MOV file, 0.1 MB. Copyright © 2018 Heinrich et al. 2018 Heinrich et al. This content is distributed under the terms of the Creative Commons Attribution 4.0 International license.

10.1128/mBio.02290-17.8 MOVIE S4  No exchange of fluorescent proteins in Vero cell aggresomes. Shown is photobleaching of the perinuclear inclusion in a Vero cell formed by cDNA expression of G250. Cells were imaged for 3 frames before and 148 frames after photobleaching (28 frames per s). No FRAP was observed. Download MOVIE S4, MOV file, 0.6 MB. Copyright © 2018 Heinrich et al. 2018 Heinrich et al. This content is distributed under the terms of the Creative Commons Attribution 4.0 International license.

Phase-separated cellular compartments demonstrate exchange between the “fluid” inclusion and dispersed “gas” cytoplasmic phase. To test whether viral proteins reversibly exchange between viroplasm and a low-concentration cytoplasmic pool, we photobleached entire viral inclusions in VSV-eGFP-P-infected cells and observed full recovery of eGFP fluorescence over 5 min in a microtubule-independent manner (Fig. 3b). As expected, this was slower than the 1 min required for internal diffusion (Fig. 3a). Quantification of multiple photobleach experiments confirmed that microtubule depolymerization did not affect FRAP kinetics of photobleached inclusions (Fig. 3c). Our data indicate rapid internal exchange of viroplasm components that reversibly exchange with a cytoplasmic pool in a microtubule-independent manner.

The dynamic properties of the viroplasm predict that exchange between discrete inclusions should also occur. When cells are coinfected with viruses expressing RFP-tagged and eGFP-tagged P, the replication compartments contain a mixture of fluorescent P proteins (see Movie S5 in the supplemental material). Such mixed viroplasm was also observed following fusion of individual cells that contained solely eGFP-P- or RFP-P-marked inclusions, even when ongoing protein synthesis was blocked by cycloheximide treatment (see Fig. S2 in the supplemental material). This result directly demonstrates the exchange (and/or fusion) between individual, already established inclusions.

10.1128/mBio.02290-17.2 FIG S2  Mixing of replication compartments following fusion of cells separately infected with rVSV-eGFP-P or rVSV-RFP-P. Syncytia were generated by fusion of Vero cells infected with either rVSV-eGFP-P (green) or rVSV-RFP-P (red) fused in the absence (upper panels) or presence (lower panels) of nocodazole to depolymerize microtubules. Yellow inclusions indicate mixed protein populations of eGFP-P and RFP-P. Cells were additionally stained for cell boundaries (WGA; wheat germ agglutinin) and nuclei (blue). Scale bars, 10 µm. Download FIG S2, TIF file, 1.2 MB. Copyright © 2018 Heinrich et al. 2018 Heinrich et al. This content is distributed under the terms of the Creative Commons Attribution 4.0 International license.

10.1128/mBio.02290-17.9 MOVIE S5  Inclusions containing both RFP-P- and eGFP-P-tagged proteins following simultaneous coinfection with rVSV-RFP-P and rVSV-eGFP-P virus. Shown is a 2D time-lapse movie of a Vero cell coinfected with rVSV-RFP-P and rVSV-eGFP-P viruses at 5 h postinfection. Inclusions containing a mixture of RFP-P- and GFP-P-tagged proteins (yellow) are observed. Inclusions are seen to undergo frequent fission and fusion events that contribute to the mixing of their content. Frame rate = 10 fps. Scale bar = 10 µm. Download MOVIE S5, MOV file, 1.7 MB. Copyright © 2018 Heinrich et al. 2018 Heinrich et al. This content is distributed under the terms of the Creative Commons Attribution 4.0 International license.

### VSV N, P, and L proteins lead to viroplasm formation.

**FIG 4:**
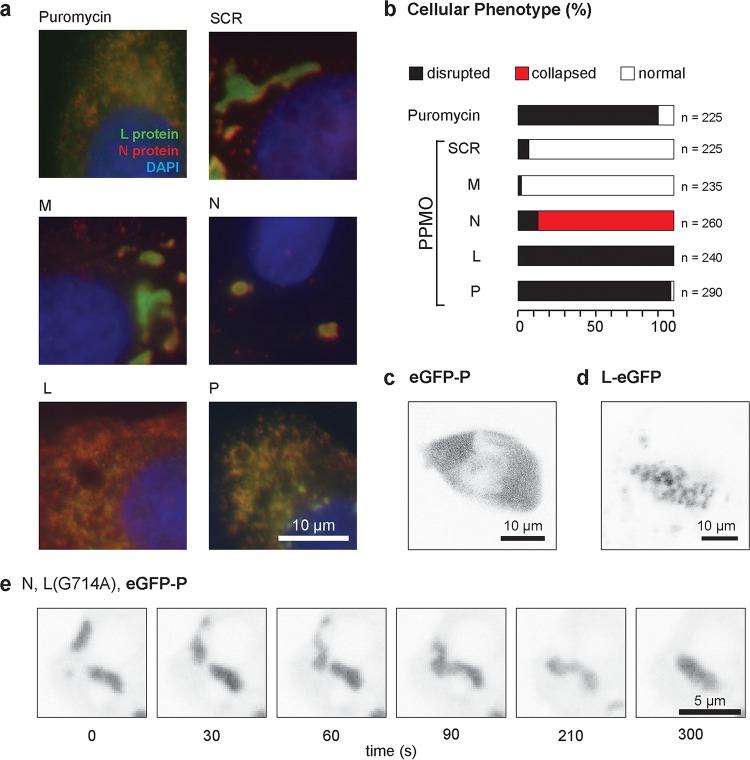
Viral replication proteins, but not replication, are required to form viroplasm in cells. (a) Representative images showing the effects on inclusion morphology in Vero cells treated with puromycin or the indicated PPMO for 3 h: VSV-L (green), VSV-N (red), and DNA (blue). Scale bar, 10 µm. (b) Classification of VSV-N and VSV-L protein staining patterns in VSV-infected Vero cells after PPMO treatment. The total cell count is indicated for each treatment. (c and d) Representative live eGFP fluorescence images of cells transfected with cDNA encoding eGFP-P (c) or L-eGFP (d). Scale bars, 10 µm. (e) Single-plane, time-lapse imaging (2 frames min^−1^) of inclusions in Vero cells transfected with the indicated plasmid cDNA encoding (N) eGFP-P and catalytically inactive L. Scale bar, 5 µm.

We took a two-pronged approach to examine the viral components that are required for formation of these liquid-like compartments. First, we either inhibited protein synthesis globally using the translation inhibitor puromycin or depleted specific viral proteins using peptide-conjugated morpholino oligomers (PPMOs) (Table 1; see Fig. S3 in the supplemental material) and investigated their effect on the cellular distribution of viral L and N by immunofluorescence microscopy of fixed cells (Fig. 4a and b). Viral protein accumulation in viroplasm is blocked by puromycin treatment of cells, indicating that active translation is required to maintain phase separation (Fig. 4a). Inhibition of *de novo* synthesis of any of the viroplasm-resident VSV protein (N, P, or L) with targeted sequence-specific PPMOs disrupted viroplasm organization, although there were subtle distinctions in the phenotype (Fig. 4b). Unlike the severe depletion phenotypes for the L and P proteins, depletion of N did not abolish phase separation but caused a rounded morphology (Fig. 4a and b). We did not test whether the N-depleted inclusions retain the fluid phase properties, although the structures would presumably still be engaged in transcription and therefore fluid. Inhibition of synthesis of M protein, a viral protein required for virus assembly, or using a control nontargeting PPMO, had no impact on viroplasm formation or appearance (Fig. 4a and b). This result implicates the involvement of the three viral proteins N, P, and L in the formation and maintenance of the compartment.

10.1128/mBio.02290-17.3 FIG S3  Specific block of viral protein synthesis using PPMOs targeted to each viral mRNA. Shown are autoradiograms of cell lysates from Vero cells treated 4 hpi with the indicated PPMO and labeled with [^35^S]MetCys for 3 h. Download FIG S3, TIF file, 0.8 MB. Copyright © 2018 Heinrich et al. 2018 Heinrich et al. This content is distributed under the terms of the Creative Commons Attribution 4.0 International license.

**TABLE 1:** PPMO sequences

Gene target	PPMO sequence^a^	VSV genome target positions
N	5′ GTAACAGA**CAT**TTTGATTACTGTT 3′	51–74
P	5′ GTGAGATTATC**CAT**GATATCTGTT 3′	1386–1409
M	5′ GGAACT**CAT**GATGAATGGATTGGG 3′	2235–2258
G	5′ GGCACTT**CAT**GGTGTCAAGGAAAC 3′	3064–3087
L	5′ TCGTGGACTTC**CAT**GATTGCTGTT 3′	4723–4746
SCR^c^	5′ AGTCTCGACTTGCTACCTCA 3′	NA^b^

^a^ Bases targeting the AUG translation start site are in boldface.

^b^ NA, not applicable.

^c^ Scrambled G.

As a complementary approach to define the viral requirements to form a fluid-like, phase-separated compartment (viroplasm), we transfected cells with expression vectors for N, P, and L individually or in combination (Fig. 4c to e). For this analysis, we took advantage of eGFP-tagged P or L expression plasmids to facilitate the visualization of the phase-separated material. Consistent with previous findings, expression of P protein alone (3) (Fig. 4d; see Fig. S4a in the supplemental material) is not associated with a phase separation of eGFP-P protein, as is also the case for N (Fig. S4b). Expression of L-eGFP alone results in the formation of cytoplasmic aggregates (Fig. 4e; Fig. S4c). When eGFP-P and -L are coexpressed, they coalesce into a distinct structure (Fig. S4d), but fluid-like viroplasm is only observed in cells expressing all 3 viral proteins: N, P, and L (see Movie S6 and Fig. S4e in the supplemental material).

10.1128/mBio.02290-17.4 FIG S4  Expression of viral N, P, and L proteins is sufficient for formation of a phase-separated compartment. Shown are representative images of cells expressing viral (a) eGFP-tagged P, (b) N, or (c) L protein. N and eGFP-P are distributed throughout the cytoplasm when expressed alone, while L forms large aggregate-like structures. Scale bars, 10 µm. (d) Colocalization (yellow) of eGFP-tagged P (green) and L (red) when coexpressed. (e) Formation of inclusion-like structures in cells coexpressing N (red), L (blue), and P. Colocalization of N and L is shown. GFP expression (green) from a negative-sense RNA replicon construct encoding GFP (green) demonstrates active ongoing viral replication and the presence of all three viral replication proteins. Scale bar, 5 µm. Download FIG S4, TIF file, 1.1 MB. Copyright © 2018 Heinrich et al. 2018 Heinrich et al. This content is distributed under the terms of the Creative Commons Attribution 4.0 International license.

10.1128/mBio.02290-17.10 MOVIE S6  Fluid inclusions are formed in Vero cells when N, eGFP-P, and L are coexpressed following cDNA transfection. A 3D time-lapse movie of a Vero cell transfected with N, eGFP-P, and L polymerase displays dynamic structures that are capable of fusion. Download MOVIE S6, MOV file, 2.8 MB. Copyright © 2018 Heinrich et al. 2018 Heinrich et al. This content is distributed under the terms of the Creative Commons Attribution 4.0 International license.

### Viroplasm formation is independent of an active RNA-dependent RNA polymerase.

We next tested whether RNA synthesis was required for formation of the phase-separated compartment or whether the N, P, and L proteins alone are sufficient. For this purpose, we exploited a catalytically inactive RNA-dependent RNA polymerase (RdRp) L mutant (G714A). Expression of the viral proteins results in formation of a liquid-like viroplasm compartment independent of their ability to drive viral RNA synthesis (Fig. 4e). This result demonstrates that the viral proteins (N, P, and L) required for replication are necessary to form a phase-separated cytoplasmic compartment in cells independent of RNA synthesis. This finding, coupled with the dissolution of inclusions following PPMO targeting of the viral N, P, and L proteins, supports a model in which the concentration of the replication machinery leads to phase separation.

## DISCUSSION

The main conclusion of our work is that viroplasm formation involves the assembly of a liquid compartment driven by the VSV N, P, and L proteins. Recent work with rabies virus using live-cell microscopy also reached this conclusion (5). In the case of rabies virus, coexpression of N and P was sufficient to form Negri body-like structures with fluid-like properties similar to those present in infected cells. For VSV, we define the N, P, and L proteins as being required for formation of similar fluid-like structures and show that neither RNA replication nor the genomic RNA is required. We further conclude through our demonstration in which viroplasm forms in a variety of mammalian, insect, and amphibian cell lines that any host requirements for viroplasm formation are conserved. Viroplasm like structures have been reported in the cytoplasm of cells infected with filoviruses (6) and paramyxoviruses (7), as well as the nucleus of cells infected with Borna disease virus (24). The conservation of similar viroplasm-like structures among representative members of different families of NNS RNA viruses suggests that the formation of phase-separated compartments is a common feature of their replication. The presence of similar inclusions in the nucleoplasm of herpesvirus-infected cells (25) raises the possibility that such liquid phase transitions may be more broadly conserved throughout the evolution of viruses.

Formation of cellular phase-separated structures is associated with low-complexity protein structures and those enriched with KH (K homology) or RRM (RNA recognition motif) RNA-binding domains (26). The VSV N and L proteins bind RNA directly, and P contains regions of low complexity (19). For VSV, expression of P alone does not result in viroplasm formation but requires the RNA binding proteins L and N. In the case of RABV, the N and P proteins alone are apparently sufficient for viroplasm formation, with the P requirements provided by the oliogomerization domain and N-RNA binding domain (5). This observation however was made in cells overexpressing RABV N and P. While we observe inclusion-like structures in cells overexpressing VSV N and P alone, maintenance of the viroplasm in infected cells requires L, as evidenced by the dissolution of structures in infected cells following the specific inhibition of L protein synthesis (Fig. 4). Overexpression studies demonstrate the requirement for L in the VSV viroplasm is independent of its RdRp activity and that the inclusion structures have a distinct morphology in the absence of L. Additional work will be required to dissect whether the different L requirement reflects intrinsic biological distinctions between RABV and VSV viroplasm or is related to differences associated with overexepression of N and P and their ability to drive formation of viroplasm independent of L.

We previously reported that VSV M protein was neither enriched nor excluded from the viroplasm structures but was rather distributed throughout the cell (3). Consistent with that earlier observation, inhibition of M protein expression using gene-specific PPMO had no effect on the formation or properties of the viroplasm (Fig. 4). Matrix protein inhibits viral gene expression (32) and plays a central role during viral particle assembly, condensing the viral RNP and facilitating budding through the membrane. It will be of significant interest to determine whether M is actively excluded from such dynamic phase-separated structures and to understand whether RNPs must first exit such structures to initiate the process of virion assembly.

The concept of the NNS RNA virus replication compartment as a liquid viroplasm presents new possibilities for thinking about the study and inhibition of viral infection. We anticipate that it will be possible to isolate molecules whose chemical properties favor partitioning into this phase-separated compartment, thereby concentrating inhibitors directly at the site of viral replication. Understanding the chemical properties of molecules that favor such partitioning may therefore aid in the development of new antiviral compounds. Efforts to purify such structures and characterize their properties have not yet been successful in NNS RNA virus-infected cells: such efforts may also be enhanced by identifying chemicals whose properties favor enrichment in these dynamic compartments. Phase-separating systems are extremely sensitive to temperature, with 1 or 2° increases being sufficient to move from a mixed to a demixed state. We would predict that lowering the temperature would decrease the fluidity of the viroplasm—a property that may aid efforts to purify them. Raising the temperature may compromise the ability of viroplasm to form, which would slow viral replication. Induction of a febrile response is a common response to viral infection, including those of NNS RNA viruses. It is tempting to speculate that such a response may also aid in control of viral replication through inhibition of such phase-separated compartments.

## MATERIALS AND METHODS

### Cell culture and manipulation.

Vero and BsRT7 cells were maintained in Dulbecco’s modified Eagle’s medium (DMEM; Life Technologies, Inc., Grand Island, NY) containing 10% fetal bovine serum (FBS; Tissue Culture BioLogicals, Tulare, CA) at 37°C and 5% CO_2_. Xenopus laevis S3 cells (Todd Stukenberg, University of Virginia) were cultured at ambient room conditions (18°C, no supplemental CO_2_) in 66% L-15 medium (Sigma-Aldrich) supplemented with 10% FBS, penicillin-streptomycin, and 1 mM sodium pyruvate. Viral infections of vertebrate cells were performed in DMEM containing 2% FBS supplemented with penicillin-streptomycin. Spodotera frugiperda Sf9 cells were cultured in Sf-900 medium (Life Technologies, Inc.) without addition of serum and maintained in a nonhumidified incubator at 28°C. C6-36 mosquito cells were cultured in Eagle’s minimum essential medium (EMEM; Life Technologies, Inc.) containing 10% FBS at 28°C. Cells were infected at a multiplicity of infection (MOI) of 5 with either wild-type recombinant VSV (rVSV), rVSV expressing eGFP-tagged (27) or RFP-tagged (3) P protein, or rVSV expressing GFP-tagged (28) L protein where the GFP-tagged L clone was constructed as previously described, except for the addition of a Gly-Gly linker before and after the GFP insertion site. Where indicated, cells were treated with 10 µg ml^−1^ nocodazole (Sigma, St. Louis, MO), 10 µg ml^−1^ puromycin (Sigma), or 5 µM peptide-conjugated morpholino oligomers (PPMOs), prepared at AVI BioPharma (Corvallis, OR) as previously described (29) and designed to target the mRNA of individual viral proteins (Table 1). VSV proteins were expressed from T7-driven plasmids in cells that had been infected with the vaccinia virus vTF7-3 1 h before transfection as previously described (18, 30). Transfection of nucleic acids was performed in Opti-MEM medium (Life Technologies, Inc.) using Lipofectamine 2000 (Life Technologies, Inc.) as recommended by the manufacturer.

### Immunofluorescence microscopy.

Vero or BsRT7 cells were fixed with 2% paraformaldehyde for 15 min, washed twice with 1× phosphate-buffered saline (PBS), and treated with ice-cold 100% methanol for 3 min. Cells were rinsed twice with 1× PBS and incubated in PBSAT (1× PBS, 0.1% Triton X-100, 1% bovine serum albumin [BSA]) followed by PBSA (1× PBS, 1% BSA), each for 10 min. For detection of VSV L, a rabbit polyclonal antiserum (3) was used at a 1:1,000 dilution, followed by an anti-rabbit secondary antibody conjugated to either DyeLight-549, DyeLight-649, or Alexa Fluor 488 (1:1,000) (Jackson ImmunoResearch Laboratories, Inc., West Grove, PA). VSV N protein was detected using the monoclonal 10G4 antibody, which was kindly provided by Douglas Lyles (Wake Forest University), followed by a 1:750 dilution of a secondary anti-mouse antibody conjugated to DyeLight-549 (Jackson ImmunoResearch Laboratories, Inc.). Cellular α-tubulin was detected using a 1:200 dilution of the monoclonal DM1A antibody (Sigma) and visualized with Alexa Fluor 488-conjugated secondary antibody (Life Techologies) at a 1:500 dilution. Nuclei were visualized with a 1:2,000 dilution of 4′,6-diamidino-2-phenylindole (DAPI; Invitrogen). Cellular membranes were stained using 10 µg ml^−1^ Alexa Fluor 647-labeled wheat germ agglutinin (WGA; Molecular Probes). Cells were mounted onto slides using ProLong Gold antifade mounting medium (Molecular Probes). Wide-field images were acquired using a Zeiss Axioplan 2 inverted fluorescence microscope (Carl Zeiss, Inc. MicroImaging, Germany). Samples were excited with a xenon lamp, and filtered emission photons were collected with a Hamamatsu Orca-HR (C4742-94) camera (Hamamatsu, Bridgewater, NJ). Confocal images were acquired using a Zeiss Observer Z1 microscope (Carl Zeiss, Inc., MicroImaging) fitted with a confocal spinning disk unit (Yokogawa Electric Corporation, Atlanta, GA) and a 63× (NA 1.4) objective. Excitation wavelengths were 473 nm for Alexa Fluor 488, 561 nm for DyeLight-549, and 660 nm for DyeLight-649.

### Cell fusion assay.

BsrT7 cells were infected (MOI of 5) with either rVSV-RFP-P or rVSV-eGFP-P. At 4 h postinfection (hpi), cells were treated with 10 µg ml^−1^ puromycin (Sigma). At 4.5 hpi, cells infected with rVSV-RFP-P were lifted from their dish by treatment with 50 mM EDTA (Sigma), to leave surface-expressed G protein intact. The cells were collected by centrifugation at 1,000 rpm for 5 min, resuspended in DMEM supplemented with 2% FBS and 10 µg ml^−1^ puromycin, overlayed onto the cell population infected with rVSV-eGFP-P, and allowed to settle for 20 min. The medium was then gently removed, and fusion buffer (10 mM Na_2_HPO_4_⋅7H_2_O, 10 mM HEPES, 10 mM morpholineethanesulfonic acid [MES], pH 5.8) was added at room temperature for 2 min. The fusion buffer was then replaced by DMEM containing 2% FBS, and cells were incubated at 37°C for 1.5 h. For imaging, cells were fixed with 2% paraformaldehyde for 15 min, washed twice with PBS (137 mM NaCl, 2.7 mM KCl, 100 mM Na_2_HPO_4_, 2 mM KH_2_PO_4_), and immunostained to visualize nuclei and cell membranes.

### Live-cell imaging.

Imaging of early infection was performed on a Nikon TE2000E microscope in a climate-controlled chamber and equipped with a Prior Proscan II motorized stage, metal halide illuminator, ORCA-ER cooled charge-coupled device (CCD) camera (Hamamatsu), 60× (PA, Ph3 objective) and controlled by Metamorph software. Image stacks were analyzed in FIJI, cells were manually outlined, and the histogram function was used to calculate the pixel distribution at each time point.

Three-dimensional confocal image stacks were acquired using a Zeiss observer Z1 microscope (Carl Zeiss, Inc., MicroImaging, Inc., Germany) fitted with a confocal spinning disk unit (Yokogawa Electric Corporation, Atlanta, GA) and a 63× (NA 1.4) objective. Excitation wavelengths were 473 nm for eGFP and 561 nm for RFP. For three-dimensional (3D) acquisitions, images were captured at intervals of 0.37 µm. The x, y, z positions of the stage were controlled using a PZ-2000 automated stage (Applied Scientific Instrumentation, Eugene, OR). Slidebook 4.2 software (Intelligent Imaging Innovations, Denver, CO) was used to acquire images and generate movies and image stacks for cropping and editing in FIJI (31).

### Inhibition of individual viral proteins and metabolic labeling.

Vero cells were plated on 12-mm coverslips placed in a 16-well dish designated for the immunofluorescence portion of this experiment. Alternatively, Vero cells were plated on 60-mm dishes designated for metabolic labeling. All cells were infected with rVSV at an MOI of 5. At 4 hpi, either 10 µg ml^−1^ puromycin (Sigma) or 5 µM PPMO designed to inhibit the translation of individual viral proteins was added. In the subset of cells designated for metabolic labeling, DMEM lacking l-methionine and l-cysteine (Life Technologies, Inc.) was supplemented with 17.5 µCi of [^35^S]-EasyTag express (PerkinElmer). At 7 hpi, cells were either fixed using 2% paraformaldehyde and prepared for immunofluorescence microscopy, or cells were lysed using lysis buffer (1% Nonidet P-40, 66 mM EDTA, 10 mM Tris-HCl, pH 7.4) and lysates analyzed by SDS-PAGE. Total cytoplasmic proteins were resolved by 10% SDS-PAGE and detected by phosphorimage analysis using a Typhoon 9400 Phosphorimager (GE Healthcare, Waukesha, WI).

### FRAP of viral inclusions.

Vero cells plated in 24-well glass-bottom plates and infected with virus or transfected with cDNA vectors, mounted in a Tokai-Hit stage-top incubation system set to 5% CO_2_ and 37°C, and imaged using a Nikon Ti-E with Perfect Focus controlled by Nikon Elements acquisition software. Images were acquired using ApoPlan 60× (NA 1.4) objectives and a Nikon A1R confocal scanner. After the acquisition of 3 time points, target cells were photobleached using a 488-nm laser and imaged. The fluorescence intensity of viral inclusions was integrated using FIJI (31), normalized for background photobleaching, and fit to a recovery curve using Kaleidegraph 4.1 (Synergy Software).
