# Supplementary figures and images for: Phase Transitions Drive the Formation of Vesicular Stomatitis Virus Replication Compartments
Source: mBio. 2018 Sep 4;9(5):e02290-17. doi: 10.1128/mBio.02290-17 (PMC6123442; doi:10.1128/mBio.02290-17)

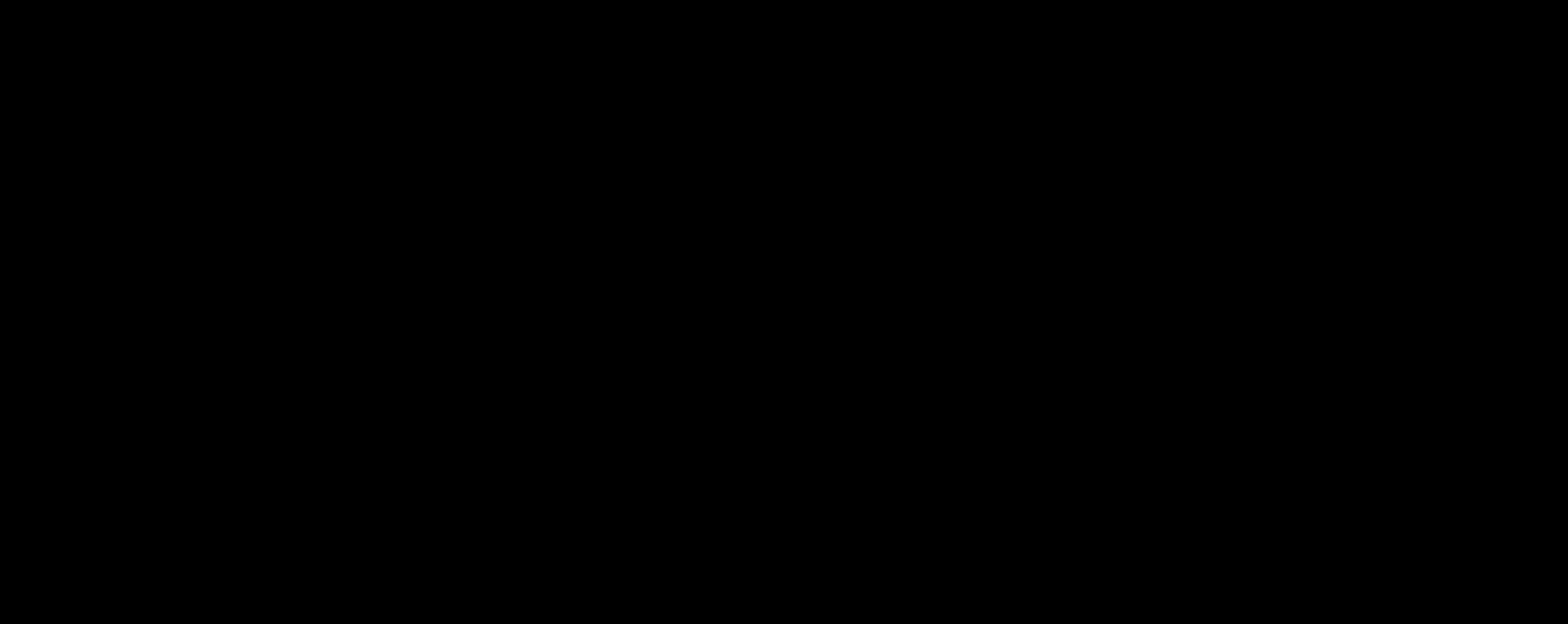

Supplement: FIG S1 [file mbo004184051sf1.tif]

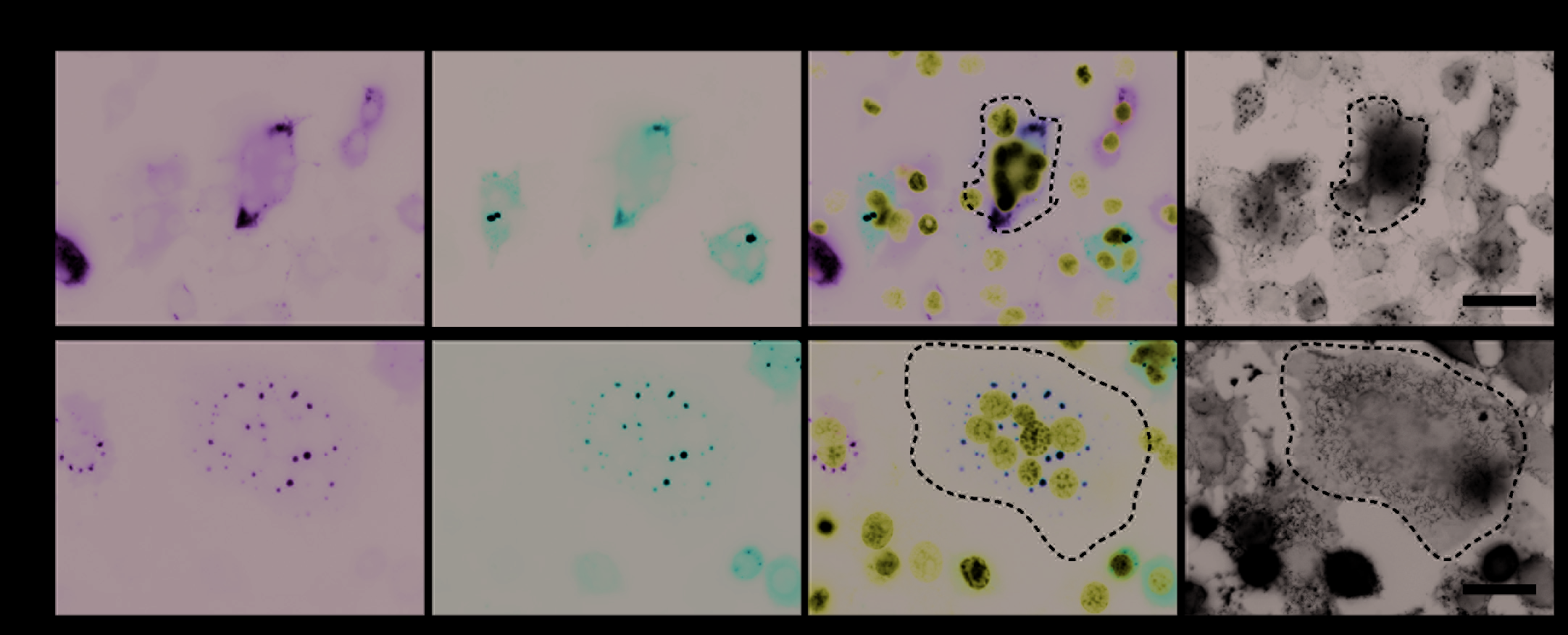

Supplement: FIG S2 [file mbo004184051sf2.tif]

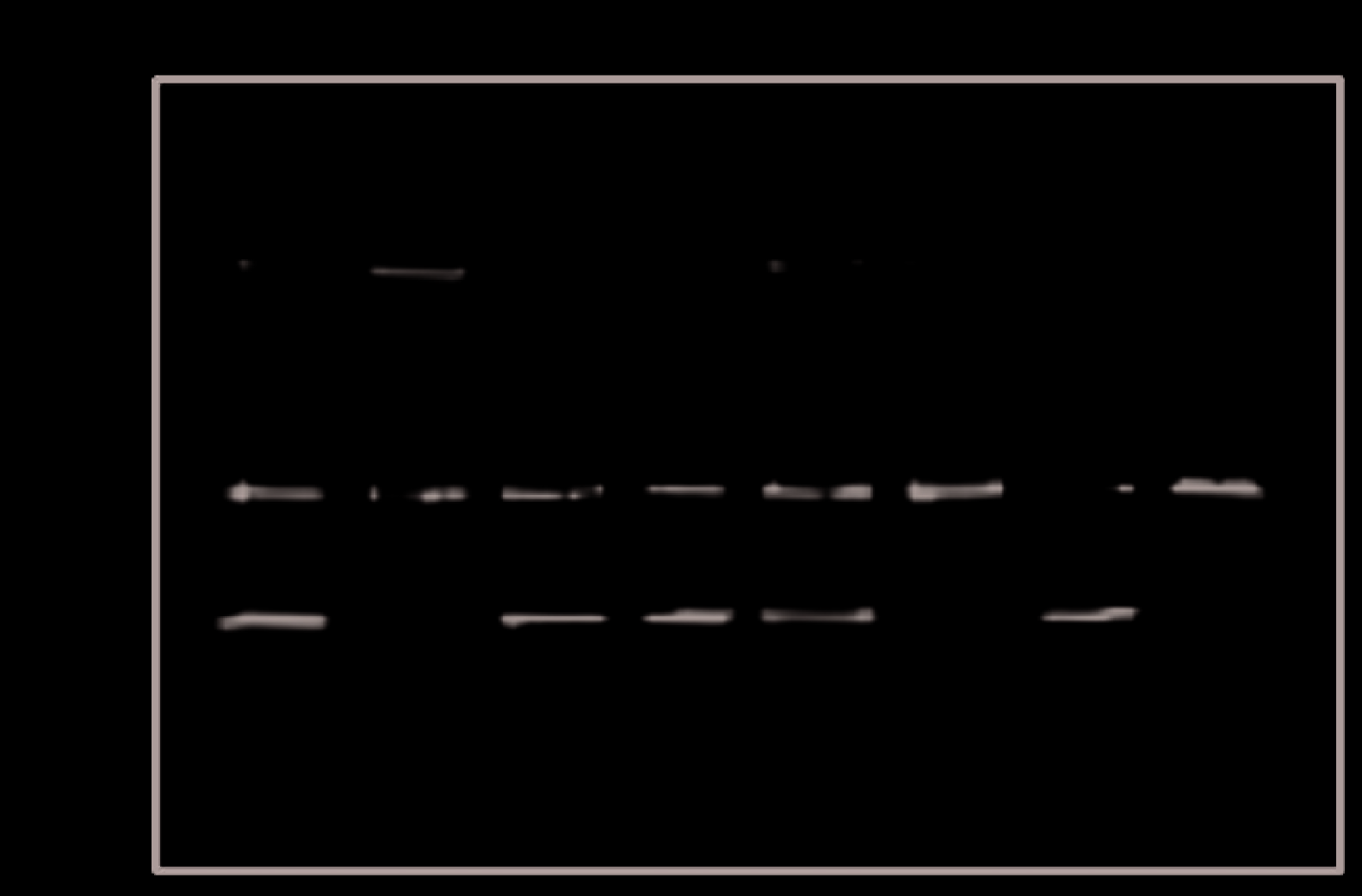

Supplement: FIG S3 [file mbo004184051sf3.tif]

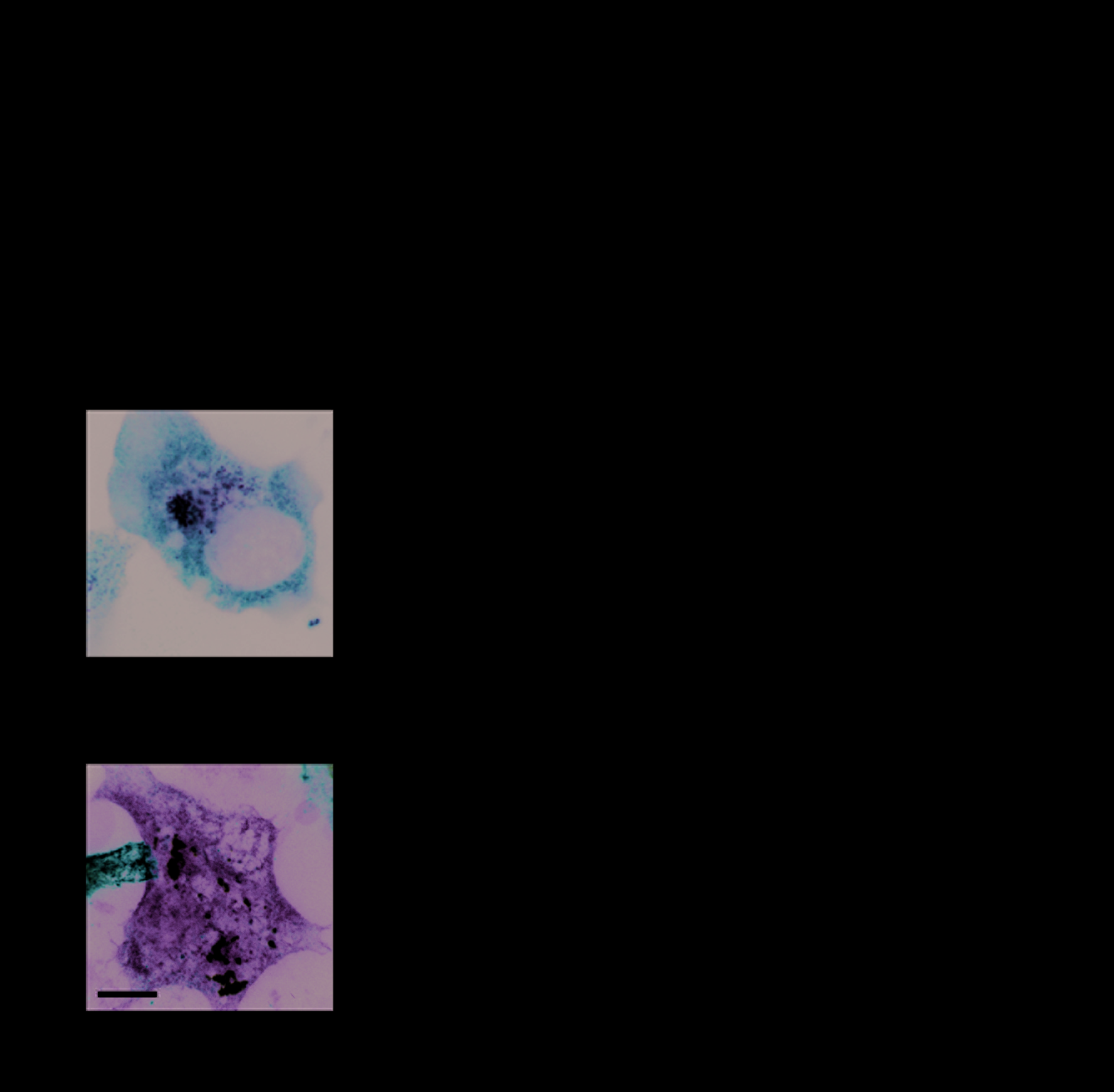

Supplement: FIG S4 [file mbo004184051sf4.tif]
